# Early B-cell factors involve in the tumorigenesis and predict the overall survival of gastric cancer

**DOI:** 10.1042/BSR20210055

**Published:** 2021-06-28

**Authors:** Qing Wang, Jiahong Liang, Xianyu Hu, Songgang Gu, Qiaodong Xu, Jiang Yan

**Affiliations:** 1 Department of Biliary-Pancreatic Minimally Invasive Surgery, The First Affiliated Hospital of Shantou University Medical College, Shantou 515041, Guangdong, China; 2 Department of General Surgery, The First Affiliated Hospital of Anhui Medical University, Hefei 230022, Anhui, China

**Keywords:** cell cycle, Early B-cell factors, gastric cancer, immune, immunotherapy, prognosis

## Abstract

Gastric cancer (GC) is a heavy health burden around the world, which is the fifth most frequent tumor and leads to the third most common cancer-related deaths. It is urgent to identify prognostic markers as the guideline for personalized treatment and follow-up. We accessed the prognostic value of Early B-cell factors (EBFs) in GC. A total of 415 GC tissues and 34 normal tissues from The Cancer Genome Atlas Stomach Adenocarcinoma (TCGA-STAD) cohort, 616 external patients from GSE15459, GSE22377, GSE51105, GSE62245 were enrolled for analysis. Univariate and multivariate Cox regression analyses were employed to evaluate the sole and integrative prognostic value of EBFs, respectively. Genetic alterations, DNA methylation of EBFs were also evaluated, as well as the involved signaling pathways. We revealed that increased EBFs associated with the poor prognosis of GC patients, the prognostic model was established in TCGA-STAD cohort, and validated in Gene Expression Omnibus (GEO) cohorts, with effectiveness in both HER2 positive and negative patients. DNA methylation was involved in the impact on prognosis. Cell cycle, immune-associated, and MAPK pathways were influenced by EBFs. Anti-CTLA4 immunotherapy is more suitable for EBFs determining high-risk groups, but not anti-PD-1/PD-L1 therapy. 5-Fluorouracil, methotrexate, vorinostat are suitable to inhibit the function of EBFs. Our new findings provide novel insight into the prediction of prognosis and clinical treatment of GC patients based on EBFs.

## Introduction

Gastric cancer (GC) is a heavy health burden around the world, which is the fifth most frequent tumor and leads to the third most common cancer-related deaths [1]. The incidence of GC is approximately two times in men than women, and with a diverse incidence around the world, the highest incidence of GC is observed in East Asia, Eastern Europe and South America [1]. In China, GC is the second most diagnosed and causes cancer-related deaths by malignancy tumor with ∼680 thousand new cases and 340 thousand new deaths per year, which is just a little less than lung cancer [2]. *Helicobacter pylori* infection, old age, cigarette smoking, alcohol consumption, family history, pernicious anemia are risk factors for the tumorigenesis of GC [3–5]. The clinical symptoms for GC patients are not significant in the early stage, therefore, most patients are diagnosed at the advanced tumor stage, which leads to a 5-year survival as low as ∼10% [6]. The Laurén classification distinguishes intestinal type, diffuse type, and unclassifiable type [7]. Intestinal type is mostly diagnosed in patients older than 70 years, along with a better prognosis [8,9]. The diffuse type often occurs in younger women, with a poor prognosis [9]. While the residual unclassifiable type has the worst prognosis because of the invasive and metastatic characteristics [10]. After reviewing 18441 GC patients, Zhao et al. [11] revealed that the independent risk factors for poor overall survival (OS) are old age, weight loss, smoking history, and process to advanced tumor stage. Minami et al. [12] found that among patients who underwent curative resection, ever-smoking, earlier age started smoking are tightly associated with the cancer-related deaths of GC; lifestyle adjustment could improve survival results. Therefore, it is urgent to identify prognostic markers as the guideline for personalized treatment and follow-up.

Traditionally, the epithelial cell features of GC are the key components for the classification of histological types. In recent years, the microenvironment infiltrated immunocytes and intratumoral stroma, showing the potential prognostic value for the progress of tumors [13–15]. Xu et al. [16] revealed that the high level of lymphocyte–monocyte ratio (LMR) and high tumor-associated macrophage (TAM) infiltration in GC patients after radical resection, and the LMR and TAM are both independent prognostic factors for OS and recurrence-free survival of GC patients. Zhou et al. [17] proposed a new immune molecular classification of GC, the immune activation subtype indicating the best OS outcome and could response to anti-PD-L1 treatment. Hu et al. [18] revealed that the regulatory T cells (Tregs) are significantly associated with the prognosis of GC patients, and established a 5-gene signature to predict the prognosis, including LRFN4, ADAMTS12, MCEMP1, HP, and MUC15. Liu et al. [19] evaluated the immune checkpoints in GC, they reported that PD-1 expression was associated with better prognosis of GC, and PD-L2 expression was related with worse survival. Mutations of PIK3CA and TP53 significantly correlated with PD-1/PD-L1/PD-L2 axis.

Early B-cell factors (EBF1–4) belong to a small family of HLH transcription factors that contain a non-basic HLH dimerization domain and an atypical zinc-finger DNA-binding domain [20]. The four EBFs express and play pivotal roles in the development of multiple cells, including B cells, adipocytes, neuronal cells [21–23]. Shen et al. [24] reported that the EBF1 could up-regulate the expression of PNO1 and promote colorectal cancer through inhibiting P53 signaling pathway. Mao et al. revealed the antitumor function of miR-204-5p through inhibiting the oncogene EBF2 in osteosarcoma [25]. Rodger et al. [26] found promoter hypermethylation and body hypomethylation of EBF3 in metastatic tumors. However, the function of EBFs in GC patients has not been evaluated till now. In the current study, we evaluated the integrated prognostic value of four EBFs in GC patients.

## Methods

### Collection of GC patient cohorts

A total of 415 GC tissues and 34 normal tissues from The Cancer Genome Atlas Stomach Adenocarcinoma (TCGA-STAD) cohort was enrolled from The Cancer Genome Atlas (TCGA) project (https://www.cancer.gov), data of transcription profiling (mRNA SeqV2), genetic alterations (gene level) and methylation (Methylation450k) for EBFs were all obtained from TCGA project. Furthermore, another 616 GC patients were also enrolled from Gene Expression Omnibus (GEO, http://www.ncbi.nlm.nih.gov/geo/), GSE15459 (*n*=197), GSE22377 (*n*=43), GSE51105 (*n*=93), and GSE62245 (*n*=283).

### Predicting the immune infiltration of GC patients

To assess the infiltration of immunocytes in GC patients, we employed the single-sample gene set enrichment analysis (ssGSEA) [27,28], implemented in the GSVA R package, to calculate the normalized enrichment score (NES) of the 28 immunocytes. The 28 gene sets of immune cell markers were previously reported by Charoentong et al*.* [29].

### Gene expression’s effects on prognosis

The mRNA expression of EBFs in normal and tumor with different stages and grades was obtained from the UALCAN (http://ualcan.path.uab.edu/index.html) [30]. Combined prognostic value of four EBFs was evaluated by SurvExpress (http://bioinformatica.mty.itesm.mx:8080/Biomatec/SurvivaX.jsp), multivariate Cox analysis was performed to generate the coefficient of each gene, which represents its weight on the prognosis of OS, and then patients could obtain the risk score through combining the weight and expression of each EBF. Another online tool, the Kaplan–Meier plotter Gastric Cancer platform (https://kmplot.com/analysis/index.php?p=service&cancer=gastric) was employed to confirm the prognostic value of EBFs in four GEO cohorts.

### Cell culture

MGC-803 and AGS cell lines were purchased from the American Type Culture Collection (ATCC, Manassas, VA), and cells were cultured in RPMI-1640 medium with 10% fetal bovine serum, 1% penicillin G and streptomycin (Gibco, Grand Island, NY, U.S.A.). All cells were maintained in a humidified 5% CO_2_ environment at 37°C. The EBF4 knocking down lentivirus was purchased from RiboBio (Guangzhou, China).

### Western blot

Cells were lysed by lysis buffer on ice, proteins (30–50 mg) were separated on 10% sodium dodecyl sulfate/polyacrylamide gel electrophoresis, then transferred to polyvinylidene difluoride membranes (Millipore, Billerica, MA). Membranes were blocked by 5% bovine serum albumin (Sigma–Aldrich) for 1 h at room temperature and then incubated with proper dilution of primary antibodies overnight at 4°C and then horseradish peroxidase-conjugated secondary antibodies. Subsequently, the ECL system (Thermo Fisher Scientific, Rochester, NY) was used for binding. The information of primary antibodies listed as follows: EBF4 antibody (Thermo Fisher; Catalog #PA5-40626), GAPDH antibody (Thermo Fisher; Catalog # PA1-987).

### Cell proliferation and invasion assay

The situation of cell proliferation was detected by MTT assay. For MTT assay, cells were seeded in 24-well plates and stained at an indicated time point using 100 ml MTT dye (Sigma) for 4 h at 37°C, then the culture medium was removed, and 150 ml dimethyl sulfoxide (Sigma) was added. The optical density at 450 nm was detected by a multilabel plate reader.

The transwell chamber obtained from Corning Costar (New York, U.S.A.) was used to detect cell invasion. The chamber was precoated with matrigel (BD Biosciences, U.S.A.) for 2 h at 37°C. Cells were collected, counted with serum-free media, and plated into the upper chambers at the density of 5 × 10^5^/ml. Then 750 ml medium with 20% FBS was added into the lower chambers and incubated for 12 h. Then cells invading the bottom of the membranes were permeabilized by methanol and stained with 0.1% Crystal Violet. The invaded cells were counted in five randomly chosen microscopic fields (100×) in each experiment and averaged for quantification.

### Genetic alteration and DNA methylation effects on prognosis

The genetic alterations of EBFs were illustrated by the cBioPortal platform (http://www.cbioportal.org/), OncoPrinter was employed to determine the frequency of genetic alterations among the recorded databases on cBioPortal [31,32]. SMART database (http://www.bioinfo-zs.com/smartapp/) was used to evaluate the association between DNA promoter region methylation and gene expression [33]. We also characterized the methylation effect on OS. The prognosis predictive value of DNA methylation CpG sites on EBFs were analyzed by a TCGA database-based methylation prognosis visualization web tool, MethSurv (https://biit.cs.ut.ee/methsurv/) [34].

### EBFs affected signaling pathways in GC

The different expression genes (DEGs) between EBFs divided into high- and low-risk groups were extracted by the limma package, |log2(fold change)| > 3 and *P*-value <0.05 was set as the cut-off value. The activated signaling pathways were revealed by the annotation of DEGs by Metascape (http://metascape.org) [35].

### Implications of immunotherapy and chemotherapy

Submap method from GeneParttern was used to predict the response to immunotherapy. Along with the published articles, we collected the gene expression profile and clinical results of 47 melanoma patients and 248 bladder cancer patients [36–39]. We also predicted the chemotherapeutic response to EBFs based on the largest publicly available pharmacogenomics database, the Genomics of Drug Sensitivity in Cancer (GDSC), with the use of GSCA online website [40].

### Statistical analysis

K–M survival analysis was used to indicate the different clinical outcomes of subgroups. Comparisons of continuous data between two groups were performed by the Student’s *t* test or Wilcoxon’s test. Pearson correlation coefficient test was employed to assess the relationship between two factors. A two-sided *P*-value <0.05 was considered statistically significant. All analyses were performed by R version 3.6.5 (http://www.r-project.org).

## Results

### EBFs were positively associated with immune infiltration in GC

**Figure 1:**
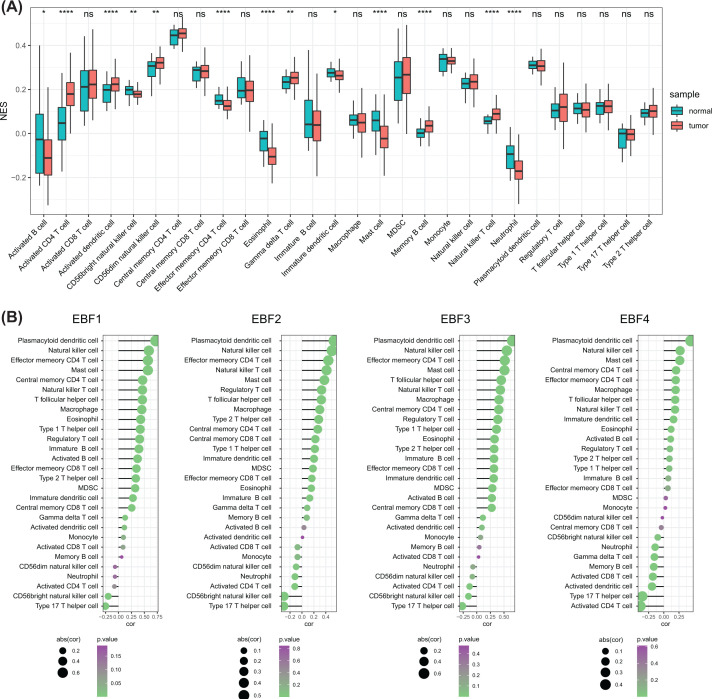
EBFs were positively associated with the infiltration of immunocytes in GC patients (**A**) The different infiltration of 28 immunocytes between GC tissues and normal stomach tissues; *, *P<*0.05; **, *P<*0.01; ***, *P<*0.001; ****, *P<*0.0001. (**B**) Correlation of EBFs and the infiltration of 28 immunocytes. The circle size represents the absolute value of the correlation; ns, no significant differences; cor, correlation; abs(cor), the absolute value of correlation.

Several studies reported immune infiltration and immunotherapy in GC patients. Based on the ssGSEA of the infiltration of 28 immunocytes, we revealed that the increased abundance of several immunocytes in the tumor microenvironment, including activated CD4 T cells, activated dendritic cells, CD56dim natural killer cell, γδ T cells, memory B cell, and natural killer T cell, while the infiltration of other types decreased in tumor tissue, including activated B cell, CD56bright natural killer cell, effector memory CD4 T cell, eosinophil, immature dendritic cell, mast cell, and neutrophil (all *P<*0.05, Figure 1A). It is reported that EBFs play pivotal roles in human and mouse immune system. Therefore, we further assessed the association of the 28 immunocytes and the expression of 4 EBFs. We observed that most of the immunocytes positively correlated with the expression of EBFs, with the consistent results of plasmacytoid dendritic cells, natural killer cells, effector memory CD4 T cells, mast cells, and the details are shown in Figure 1B and Supplementary Table S1.

### High expression of EBFs linked with advanced tumor stage and poor prognosis

**Figure 2:**
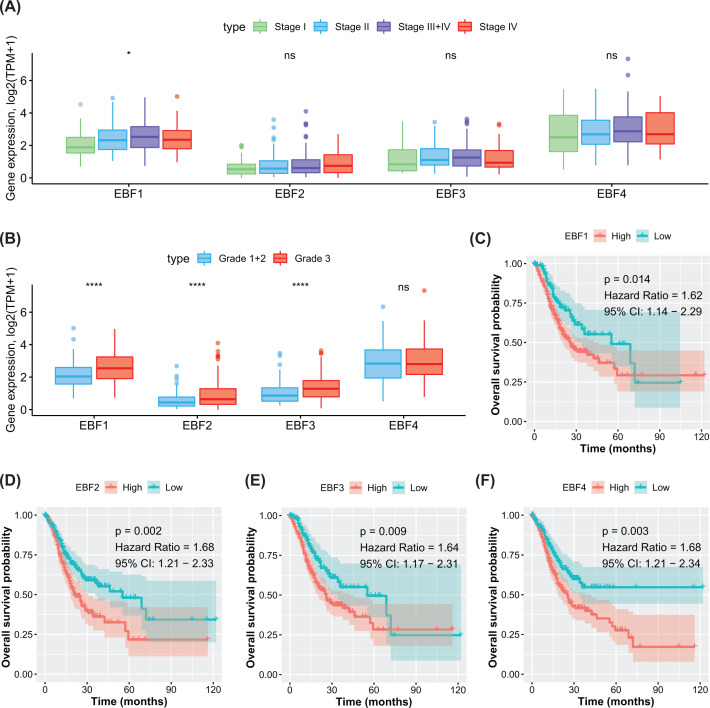
High expression of EBFs linked with advanced tumor and poor prognosis (**A**) The variable expression of EBFs in different tumor stages; statistical significance calculated by one-way anova test; TPM, transcripts per million. (**B**) The variable expression of EBFs in different tumor grades; statistical significance calculated by one-way anova test. (**C–F**) Poor prognosis was observed in the high-expression group of EBF1, EBF2, EBF3, and EBF4. The groups of high- and low-expression were separated by the median value of gene expression. ns, not significant; *, *P* <0.05; ****, *P* <0.0001.

The mRNA expression of EBFs in GC patients was evaluated among different stages and grades of tumors. We observed the increasing tendency of EBFs in the advanced tumor stage for four EBFs, but only EBF1 showed the statistic difference (*P<*0.05, Figure 2A). As for tumor grade, we combined Grades 1 and 2 and observed that the expression of EBF1, EBF2, and EBF4 increased in Grade 3 tumors, in comparison with Grades 1 and 2 (all *P<*0.001, Figure 2B). Furthermore, we separated the patients into two groups by the median value of EBFs expression. The high level of EBF1 (HR = 1.62, 95% CI = 1.14–2.29, *P*=0.014, Figure 2C) related to the poor prognosis of GC patients, as well as EBF2 (HR = 1.68, 95% CI = 1.21–2.33, *P*=0.002, Figure 2D), EBF3 (HR = 1.64, 95% CI = 1.17–2.31, *P*=0.009, Figure 2E), and EBF4 (HR = 1.68, 95% CI = 1.21–2.34, *P*=0.003, Figure 2F).

### Knocking down the expression of EBF4 can inhibit cell proliferation and invasion

**Figure 3:**
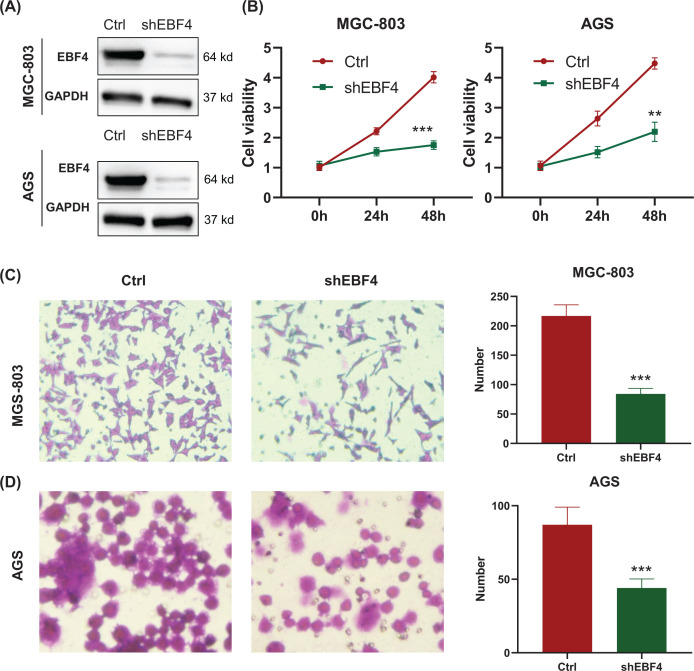
Knockdown of EBF4 inhibited the proliferation and invasion of GC cells (**A**) Confirming the knockdown efficacy of EBF4 by Western blotting. (**B**) Knockdown of EBF4 inhibited cell proliferation in both MGC-803 and AGS cells. (**C,D**) Knockdown of EBF4 inhibited cell invasion in both MGC-803 and AGS cells. The pictures of cell invasion were recorded by 100× magnification. Bar plots in the right side are the quantitated invaded cell counts in control and shEBF4 groups. The differences in two groups were evaluated by Student’s *t* test. **, *P<*0.01; ***, *P<*0.001.

We found that the high level of EBF4 was associated with the poor prognosis of GC patients. To further confirm the finding, we altered the expression of EBF4 to see the phenotype changing. We successfully knocked down the expression of EBF4 by shEBF4 lentivirus in both MGC-803 and AGS cell lines (Figure 3A), then we recorded the cell proliferation status in control group and shEBF4 group by MTT assay at the time of 0, 24, and 48 h. We observed that the knock down of EBF4 led to the inhibition of cell proliferation in both MGC-803 and AGS cell lines (*P<*0.05, Figure 3B). Moreover, we also evaluated the invasion ability with or without EBF4 knockdown by transwell assay. Knowing down EBF4 can weaken the cell invasion function in both MGC-803 and AGS cell lines (*P<*0.05, Figure 3C,D).

### High integrative risk score of four EBFs linked with poor prognosis of GC patients

**Figure 4:**
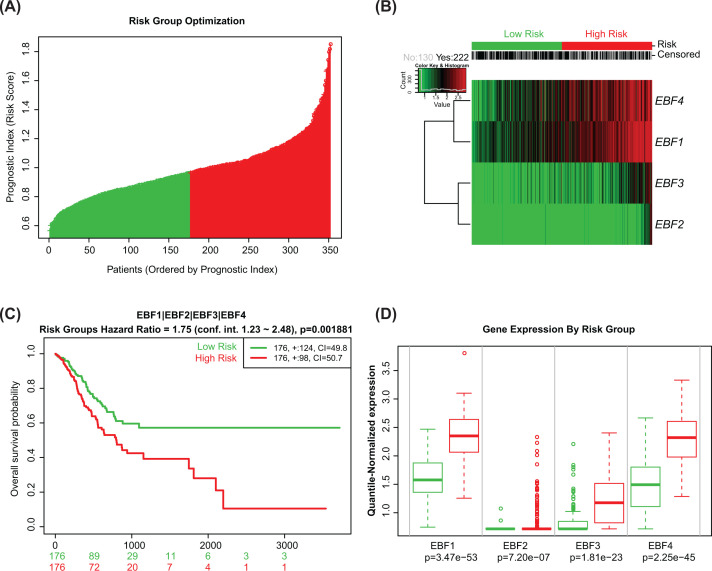
Integrative prognostic value of four EBFs by the multivariate Cox regression (**A**) Risk scores of patients calculated based on the coefficient and expression of EBFs. Patients were separated into high- and low-risk groups by the median value of risk score. (**B**) Heatmap showing the expression distribution of four EBFs in high- and low-risk patients. (**C**) Integrative prognostic value of four EBFs among high- and low-risk patients. (**D**) Expression of EBF1, EBF2, EBF3, and EBF4 in high- and low-risk groups. The difference expressions of four EBFs in high- and low-risk groups were calculated by Student’s *t* test.

To obtain the integrative risk score, we used the multivariate Cox regression analysis to calculate the coefficients of EBFs (Table 1), then we calculated the risk score of each patient, with the median risk score as the cut-off value, the patients were divided into high- and low-risk groups (Figure 4A). The expression of all four EBFs in high- and low-risk groups is displayed by heatmap in Figure 4B. We observed the results that patients in the high-risk group met a poor prognosis than low-risk group (HR = 1.75, 95% CI = 1.23–2.48, *P*=0.002, Figure 4C). And we also observed that the expression of EBF1, EBF2, EBF3, and EBF4 are higher in high-risk group, in comparison with low-risk group (all *P<*0.001, Figure 4D).

**Table 1:** Coefficient of EBFs to the prognosis of STAD patients

	Co-ef	Exp (co-ef)	Se (co-ef)	z
**EBF1**	0.18173	1.19929	0.23844	0.762
**EBF2**	0.40163	1.49425	0.34885	1.151
**EBF3**	0.02409	1.02438	0.30734	0.078
**EBF4**	0.15289	1.16520	0.16305	0.938

Abbreviations: Co-ef, coefficient; Exp (co-ef), expectation (co-ef); Se (co-ef), standard error (co-ef).

### Confirming the single and integrative prognostic values of EBFs in external validation cohorts

**Figure 5:**
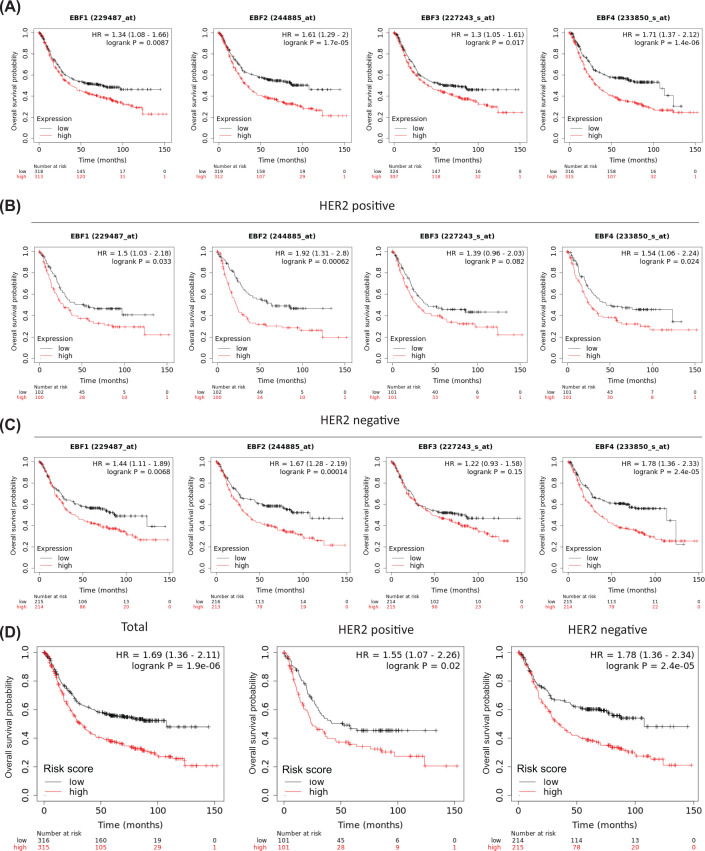
Prognostic value of EBFs in external GEO cohorts (**A**) Separate OS probabilities of patients with high- and low-expression EBFs. The groups were set by the median expression value. (**B**) The diverse OS probability of EBFs in HER2-positive patients. (**C**) The diverse OS probability of EBFs in HER2-negative patients. (**D**) Prognostic value of the four EBFs integrative risk scores of GEO cohort in total, HER2-positive and HER2-negative patients. The risk score was calculated by the TCGA-STAD determined coefficient.

On the platform of Kaplan–Meier plotter Gastric Cancer, we validated the prognostic value of EBFs among 616 patients obtained from four GEO cohorts, including GSE15459, GSE22377, GSE51105, and GSE62245. We set the median expression level of EBFs as the cut-off value, revealing that the increased expression of EBFs reflected the unfavorable prognosis, EBF1 (HR = 1.34, 95% CI = 1.08–1.66, *P*=0.009), EBF2 (HR = 1.61, 95% CI = 1.29–2.00, *P<*0.001), EBF3 (HR = 1.30, 95% CI = 1.05–1.61, *P*=0.017), and EBF4 (HR = 1.71, 95% CI = 1.37–2.12, *P*<0.001, Figure 5A), which was consistent with the new findings from the TCGA-STAD cohort. Some studies reported that HER2 positivity was associated with a significantly worse prognosis of GC patients [41,42], therefore, we assessed the prognostic value of EBFs in HER2 positive and HER2 negative patients, respectively. In the HER2-positive GC patients, high-expression EBF1 (HR = 1.5, 95% CI = 1.03–2.18, *P*=0.033), EBF2 (HR = 1.92, 95% CI = 1.31–2.80, *P<*0.001), and EBF4 (HR = 1.54, 95% CI = 1.06–2.24, *P*=0.024, Figure 5B) linked with poor prognosis; similar results were observed in HER2-negative GC patients, EBF1 (HR = 1.44, 95% CI = 1.11–1.89, *P*=0.007), EBF2 (HR = 1.67, 95% CI = 1.28–2.19, *P<*0.001), and EBF4 (HR = 1.78, 95% CI = 1.36–2.33, *P*<0.001, Figure 5C). It is interesting that the prognostic value of EBF3 was dismissed after adjusting by HER2 status, which means that EBF3 might be involved with the HER2 status. With the same factor coefficients of the four EBFs generated from TCGA-STAD cohort, we calculated the risk score of 616 patients from GEO cohort. Patients with high-risk score along with the increased risk of poor prognosis in total (HR = 1.69, 95% CI = 1.36–2.11, *P<*0.001), as well as the subgroups of HER2 positive (HR = 1.55, 95% CI = 1.07–2.26, *P*=0.02) and HER2 negative (HR = 1.78, 95% CI = 1.36–2.34, *P<*0.001, Figure 5D).

### Genetic alteration and DNA methylation of EBFs impact the prognosis of GC patients

**Figure 6:**
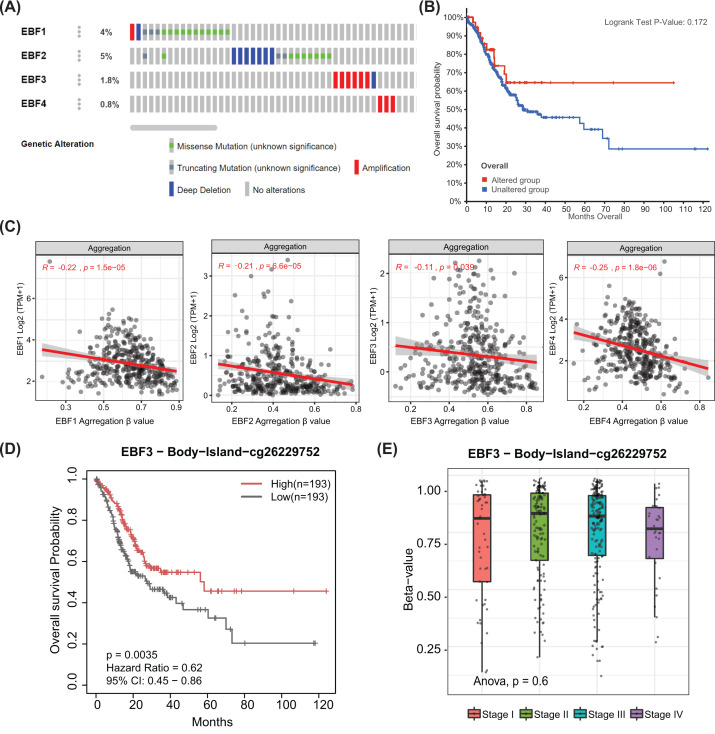
The impact of genetic alteration and DNA methylation of EBFs on GC patients’ prognosis (**A**) Oncoprinter showing the genetic mutation and copy number alteration in EBFs. (**B**) K–M plot showing the OS of GC patients in genetic altered and unaltered groups. (**C**) The correlation between promoter DNA methylation of EBFs and their mRNA expression. (**D**) Lower DNA methylation β-value of EBF3 cg26229752 associated with the poor prognosis. (**E**) DNA methylation β-value of EBF3 cg26229752 decreased in advanced stages. Beta values (β) are the estimate of methylation level using the ratio of intensities between methylated and unmethylated alleles. β are between 0 and 1 with 0 being unmethylated and 1 fully methylated.

It is reported that genetic alteration and DNA methylation can impact the clinical outcome of tumors through epigenetics, therefore, we assessed them in EBFs expression and GC patients prognosis. We observed approx. 0.8–5% genetic alteration among the four EBFs, more missense mutation was observed in EBF1, while deep deletion and missense mutation both appeared in EBF2 (Figure 6A). However, we did not find the impact of EBFs genetic alteration to OS of GC patients (*P*=0.172, Figure 6B). As for DNA methylation, we evaluated the prognostic value of the single CpG site of EBFs by univariate Cox regression analysis, the CpG sites with significant prognostic value are displayed in Table 2. We also evaluated the regulation of prognostic CpG sites on the mRNA expression of EBFs, it is surely that the prognostic CpG sites negatively regulated the mRNA expression of EBFs (all *P<*0.05, Figure 6C). As mentioned above, increased EBF3 reflected poor prognosis, the increased DNA methylation β-value of EBF3-cg26229752 was associated with favorable prognosis (HR = 0.62, 95% CI = 0.45–0.86, *P*=0.0035, Figure 6D). Moreover, we also observed the decreased tendency of the DNA methylation β-value of EBF3-cg26229752 in the advanced tumor stage (*P*=0.6, Figure 6E).

**Table 2:** The prognostic values of CpG in the EBFs by MethSurv

CpGs	HR	95% CI	*P*-value
EBF1-1stExon/5′UTR-N_Shore-cg 17009297	0.634	0.418–0.963	0.032
EBF1-Body-Island-cg11891579	0.605	0.398–0.918	0.018
EBF1-Body-N_Shore-cg01192077	0.612	0.403–0.93	0.021
EBF1-Body-N_Shore-cg02485328	0.664	0.468–0.943	0.022
EBF1-Body-N_Shore-cg11898646	0.443	0.281–0.697	<0.001
EBF1-Body-N_Shore-cg24903893	0.633	0.419–0.954	0.029
EBF1-Body-Open_Sea-cg04149356	0.613	0.406–0.926	0.02
EBF1-Body-Open_Sea-cg27127608	0.581	0.382–0.882	0.011
EBF1-TSS1500-Island-cg00251610	0.615	0.4–0.946	0.027
EBF2-1stExon-Island-cg22841810	0.504	0.321–0.793	0.003
EBF2-Body-Island-cg08283882	0.624	0.406–0.96	0.032
EBF2-Body-Island-cg11813976	0.599	0.427–0.842	0.003
EBF2-Body-Island-cg14346873	0.616	0.406–0.934	0.023
EBF2-Body-Island-cg17451609	0.629	0.446–0.888	0.008
EBF2-Body-Island-cg22280475	0.644	0.422–0.985	0.042
EBF2-Body-N_Shore-cg05487589	1.677	1.185–2.373	0.004
EBF2-Body-N_Shore-cg05748163	0.532	0.344–0.824	0.005
EBF2-Body-N_Shore-cg09559189	0.538	0.342–0.846	0.007
EBF2-Body-N_Shore-cg14855519	0.543	0.348–0.847	0.007
EBF2-Body-Open_Sea-cg00318347	0.632	0.414–0.966	0.034
EBF2-Body-Open_Sea-cg08322280	0.581	0.381–0.888	0.012
EBF2-Body-Open_Sea-cg10905495	0.587	0.387–0.892	0.012
EBF2-Body-Open_Sea-cg22883472	0.551	0.392–0.775	<0.001
EBF2-Body-Open_Sea-cg22987487	0.591	0.42–0.831	0.002
EBF2-TSS1500-Island-cg00424572	0.6	0.39–0.923	0.02
EBF2-TSS1500-Island-cg11006995	0.703	0.495–0.999	0.049
EBF2-TSS1500-Island-cg18506390	0.542	0.345–0.853	0.008
EBF2-TSS1500-Island-cg22524065	0.662	0.439–0.999	0.05
EBF2-TSS1500-Island-cg24295381	0.695	0.502–0.962	0.028
EBF2-TSS200-Island-cg12863565	0.657	0.433–0.997	0.048
EBF3-Body-Island-cg00040566	0.667	0.481–0.925	0.015
EBF3-Body-Island-cg05186311	0.585	0.38–0.9	0.015
EBF3-Body-Island-cg05734295	0.694	0.502–0.959	0.027
EBF3-Body-Island-cg05892817	0.588	0.382–0.903	0.015
EBF3-Body-Island-cg07525420	0.546	0.353–0.846	0.007
EBF3-Body-Island-cg09890775	0.523	0.333–0.823	0.005
EBF3-Body-Island-cg11314211	0.666	0.448–0.989	0.044
EBF3-Body-Island-cg13638229	0.636	0.459–0.881	0.006
EBF3-Body-Island-cg13916885	0.541	0.341–0.86	0.009
EBF3-Body-Island-cg14405924	0.538	0.345–0.84	0.006
EBF3-Body-Island-cg16589299	0.557	0.36–0.863	0.009
EBF3-Body-Island-cg17726092	0.593	0.388–0.905	0.016
EBF3-Body-Island-cg19424261	0.663	0.477–0.923	0.015
EBF3-Body-Island-cg22655696	0.63	0.409–0.969	0.035
EBF3-Body-Island-cg23042510	0.59	0.381–0.913	0.018
EBF3-Body-Island-cg25746778	0.626	0.41–0.956	0.03
EBF3-Body-Island-cg26229752	0.52	0.34–0.794	0.002
EBF3-Body-Island-cg26729372	0.587	0.381–0.902	0.015
EBF3-Body-N_Shelf-cg13734106	0.657	0.465–0.928	0.017
EBF3-Body-N_Shore-cg06163425	0.618	0.439–0.871	0.006
EBF3-Body-N_Shore-cg07194839	0.623	0.449–0.865	0.005
EBF3-Body-N_Shore-cg07890827	0.71	0.51–0.987	0.042
EBF3-Body-N_Shore-cg15069620	0.67	0.48–0.936	0.019
EBF3-Body-N_Shore-cg23250262	1.574	1.136–2.181	0.006
EBF3-Body-Open_Sea-cg02449166	0.63	0.446–0.889	0.009
EBF3-Body-Open_Sea-cg15229245	0.559	0.368–0.85	0.006
EBF3-Body-Open_Sea-cg15251598	0.568	0.372–0.869	0.009
EBF3-Body-Open_Sea-cg16093296	0.687	0.494–0.956	0.026
EBF3-Body-Open_Sea-cg25386707	0.621	0.407–0.949	0.028
EBF3-Body-S_Shelf-cg14250961	0.573	0.367–0.894	0.014
EBF3-Body-S_Shore-cg03906996	0.702	0.508–0.969	0.032
EBF3-Body-S_Shore-cg04217539	0.653	0.439–0.971	0.035
EBF3-Body-S_Shore-cg14986671	1.405	1.018–1.94	0.039
EBF3-TSS1500-Island-cg02700606	0.523	0.335–0.817	0.004
EBF4-3′UTR-Open_Sea-cg26576937	0.648	0.43–0.979	0.039
EBF4-Body-Island-cg05825244	0.629	0.443–0.894	0.01
EBF4-Body-Island-cg12497914	0.62	0.412–0.932	0.021
EBF4-Body-Island-cg26332061	0.682	0.48–0.97	0.033
EBF4-Body-Island-cg27226920	0.69	0.486–0.979	0.038
EBF4-Body-S_Shore-cg05857996	0.636	0.421–0.961	0.031

### EBFs promote tumorigenesis through cell cycle and immune-associated pathways

**Figure 7:**
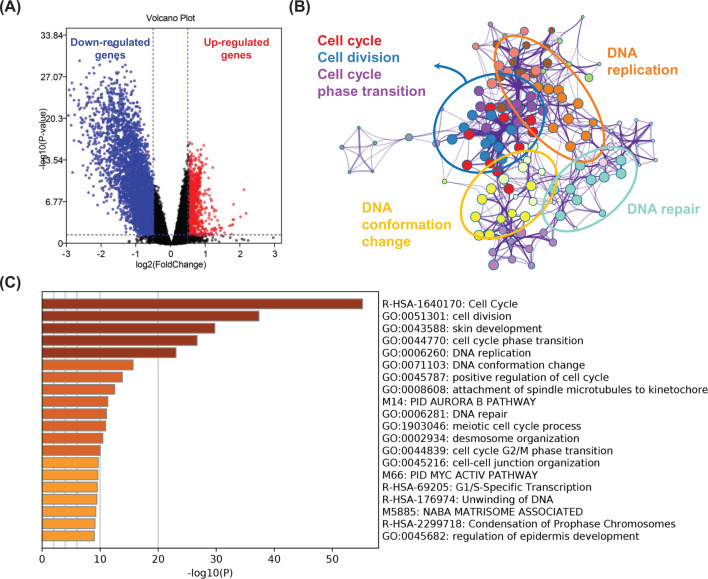
DEGs and biological process in EBFs determined high-risk group (**A**) DEGs between EBFs determined high- and low-risk groups; red points represented up-regulated genes, blue points represented down-regulated genes. (**B**) Network plot showing the relationships between the enriched biological terms. (**C**) Biological process enrichment analysis has been carried out with the following ontology sources: KEGG Pathway, GO Biological Processes, Reactome Gene Sets, and Canonical Pathways.

**Figure 8:**
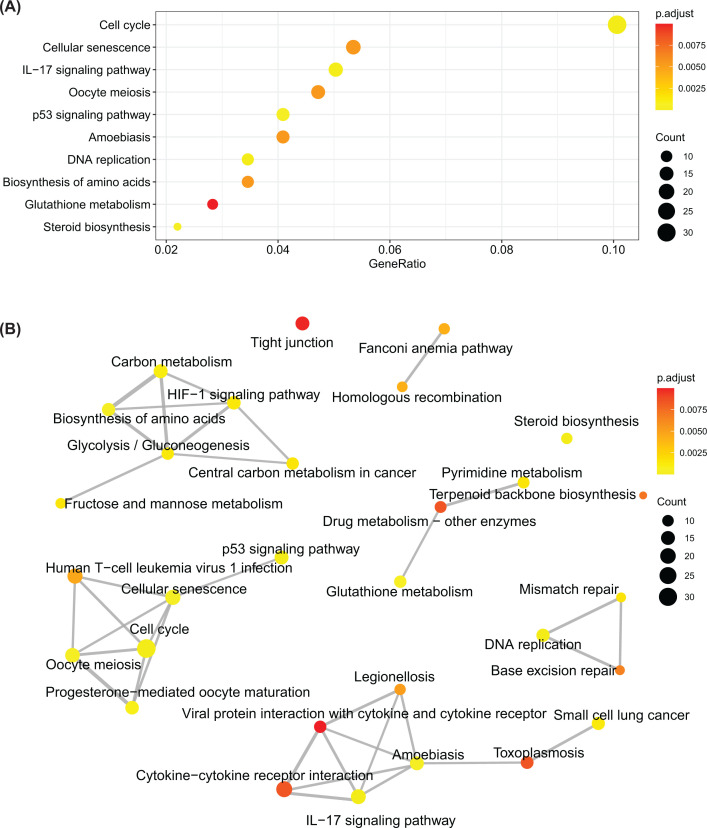
Signaling pathways and network impact via EBFs (**A**) The top 10 enriched signaling pathways of increased DEGs. (**B**) The associated network of top 30 enriched signaling pathways. The color represented the adjusted *P*-value, and the circle size inflected the gene counts in the enriched pathway.

To further investigate the biological process impacted by EBFs, we firstly obtained the DEGs between EBFs determined high- and low-risk groups (Figure 7A). The increased 765 genes were annotated by the Metascape, which revealed the activated biological processes in high-risk groups, including cell cycle, cell division, cell cycle phase transition, DNA replication, and DNA repair (Figure 7B,C). We also employed the Kyoto Encyclopedia of Genes and Genomes (KEGG) enrichment analysis, and found that the most activated signaling pathway impacted by EBFs is cell cycle (Figure 8A), consistent with the results from Metascape. Furthermore, cell cycle, cellular senescence, and p53 signaling pathways were closely related (Figure 8B).

### Anti-CTLA4 and several small molecules are effective treatments for EBF-determined high-risk group

**Figure 9:**
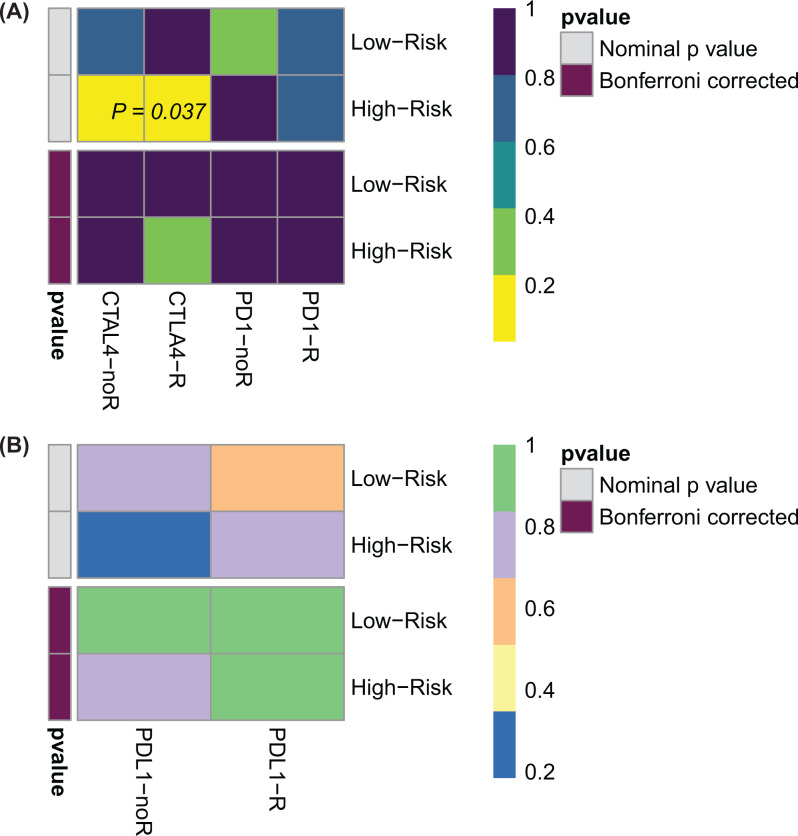
Differential putative immunotherapeutic response in low- and high-risk groups (**A**) Based on the response patients from the melanoma cohort. (**B**) Based on response patients from IMvigor210 cohort. The Submap analysis was enrolled to compare the similarity between the EBFs determined risk groups and the gene matrix of responders from immunotherapy.

To investigate the potential treatment way for GC patients, we firstly compared the gene expression profile in high- or low-EBFs determined groups with the gene expression of responders or non-responders from the melanoma cohorts mentioned above. Submap method was applied for this analysis. We revealed that patients in the high-risk group might benefit more from the anti-CTLA4 therapy, but not the anti-PD-1 therapy (Figure 9A). With the same Submap analysis, we predicted the response to anti-PD-L1 therapy as well, responders and non-responders to anti-PD-L1 in IMvigor210 bladder cancer cohort. The results in Figure 9B also indicated that there is no response to anti-PD-L1 treatment in both risk groups. Additionally, we also predicted the potential inhibitor of EBFs, causing the increased EBFs indicate poor prognosis, through the GSCA online website. We found that the increased EBFs negatively correlated with the IC_50_ of several small molecules (Table 3), which might be the potential therapeutic medicine in the treatment of GC.

**Table 3:** Prediction of the potential treatment small molecules of EBFs

Gene symbol	Drug name	Correlation	FDR
EBF1	5-Fluorouracil	−0.18	<0.01
EBF2	5-Fluorouracil	−0.1	<0.01
EBF3	5-Fluorouracil	−0.13	<0.01
EBF1	AR-42	−0.24	<0.01
EBF2	AR-42	−0.14	<0.01
EBF3	AR-42	−0.14	<0.01
EBF1	AT-7519	−0.21	<0.01
EBF2	AT-7519	−0.14	<0.01
EBF3	AT-7519	−0.12	<0.01
EBF1	CAY10603	−0.28	<0.01
EBF2	CAY10603	−0.14	<0.01
EBF3	CAY10603	−0.15	<0.01
EBF1	CUDC-101	−0.21	<0.01
EBF2	CUDC-101	−0.15	<0.01
EBF3	CUDC-101	−0.11	<0.01
EBF1	FK866	−0.25	<0.01
EBF2	FK866	−0.16	<0.01
EBF3	FK866	−0.22	<0.01
EBF4	GSK269962A	−0.1	0.02
EBF1	GSK429286A	−0.1	0.01
EBF3	GSK429286A	−0.13	<0.01
EBF1	I-BET-762	−0.29	<0.01
EBF2	I-BET-762	−0.18	<0.01
EBF3	I-BET-762	−0.14	<0.01
EBF1	Methotrexate	−0.28	<0.01
EBF2	Methotrexate	−0.12	<0.01
EBF3	Methotrexate	−0.15	<0.01
EBF1	Navitoclax	−0.27	<0.01
EBF2	Navitoclax	−0.16	<0.01
EBF3	Navitoclax	−0.27	<0.01
EBF1	PHA-793887	−0.3	<0.01
EBF2	PHA-793887	−0.11	<0.01
EBF3	PHA-793887	−0.16	<0.01
EBF1	PIK-93	−0.32	<0.01
EBF2	PIK-93	−0.1	0.01
EBF3	PIK-93	−0.18	<0.01
EBF1	QL-X-138	−0.32	<0.01
EBF2	QL-X-138	−0.11	<0.01
EBF3	QL-X-138	−0.16	<0.01
EBF1	QL-XI-92	−0.28	<0.01
EBF2	QL-XI-92	−0.11	<0.01
EBF3	QL-XI-92	−0.13	<0.01
EBF1	SB52334	−0.12	<0.01
EBF3	SB52334	−0.15	<0.01
EBF4	SB52334	−0.11	0.01
EBF1	SNX-2112	−0.22	<0.01
EBF2	SNX-2112	−0.12	<0.01
EBF3	SNX-2112	−0.11	<0.01
EBF1	THZ-2-102-1	−0.23	<0.01
EBF2	THZ-2-102-1	−0.13	<0.01
EBF3	THZ-2-102-1	−0.15	<0.01
EBF1	Tubastatin A	−0.27	<0.01
EBF2	Tubastatin A	−0.14	<0.01
EBF3	Tubastatin A	−0.22	<0.01
EBF1	UNC0638	−0.29	<0.01
EBF2	UNC0638	−0.15	<0.01
EBF3	UNC0638	−0.16	<0.01
EBF1	Vorinostat	−0.22	<0.01
EBF2	Vorinostat	−0.18	<0.01
EBF3	Vorinostat	−0.24	<0.01
EBF1	WZ3105	−0.2	<0.01
EBF2	WZ3105	−0.15	<0.01
EBF3	WZ3105	−0.16	<0.01

## Discussion

EBF family members are transcription factors, also known as Collier/Olf1/EBF proteins, express and function in diverse cells and tissues. A recent study demonstrated that EBFs and Pax5 function together to mediate the development of B-cell lineages during B lymphopoiesis, and EBFs could promote B-cell fates with the absence of Pax5 [43]. Dias et al. [44] reported the EBF1 can partially rescue the development of B cells in mice model which lacks IL17 or ZBTB17. Schwartz et al. [45] demonstrated the transcript function of EBF1 to the co-stimulatory receptor SLAMF1/CD150 in B cells, and affects the B-cell differentiation and activation. EBF2 is required for the thermogenic establishment and maintenance of brown adipocytes [46]. Moreover, EBFs act as a conserved component to regulate the expression of axonal pathfinding and neuronal differentiation associated proteins [47]. The association between EBFs and several diseases was also reported. Li et al. [48] found the rs36071027 polymorphism in EBF1 could evaluate the risk of coronary artery disease, as well as the severity. Ying et al. [49] also revealed the risk of rs987401919 and rs36071027 of EBF1 in coronary artery disease, which also interacted with smoking and alcohol abuse, impact on blood pressure and lipid contents of patients. Bae et al. [50] reported the result of EBF2 rs10866845 increased the risk of Kawasaki disease, the mucocutaneous lymph node syndrome. As for the influence of EBFs to cancers, Xu et al. [51] demonstrated the promoting function of EBF1 to USP5, the increased expression of USP5 could promote the proliferation of colorectal cancer through stabilizing the translation elongation factor. Mao et al. [52] demonstrated the positive impact of EBF3 on the progression of hepatocellular carcinoma by increasing the number of tumor cells in S phase. Fatima et al. [53] reported the copy number alteration of EBF4 links with reduced survival of breast cancer patients from the analysis of TCGA database. All these evidences prompted us to clarify whether the EBFs have any role in the tumorigenesis of GC. Unfortunately, there is no description of the prognostic value of the four EBFs in GC.

In the current study, we first evaluated the infiltration of 28 immunocytes in TCGA-STAD samples, and revealed the altered abundance of several immunocytes between tumor and normal GC tissues. Then, we assessed the association of four EBFs and immunocytes infiltration, revealing that all four EBFs are positively impacted on the infiltration of immune cells in GC patients, especially for the plasmacytoid dendritic cell, nature killer cells, effector memory CD4^+^ T cell, and mast cell. Further analysis between EBFs expression and tumor stage and clinical outcome told us that the four EBFs are an oncogene for GC tumorigenesis. The integrated prognostic value was found in TCGA-STAD cohort, and validated in both HER2 positive and HER2 negative GC patients in GSE15459, GSE22377, GSE51105, GSE62245 cohorts. The oncogene function of EBFs was studied by several *in vivo* and *in vitro* studies. Genetic alteration of EBFs might not impact on the prognosis of GC patients, but the DNA methylation of EBFs displayed a negative regulation of EBFs expression and involved into the influence of GC patients OS. Kim et al. [54] detected ∼40.4% (42/104) patients with promoter methylation of EBF3, and the promoter methylation linked with lymphatic invasion (*P*=0.013) and poor survival (*P*=0.038) in GC. Dunwell et al. [55] also observed more DNA methylation of EBF2 in the blast crisis status than the chronic phase of chronic myeloid leukemia, which lined the EBF2 DNA methylation in advanced stage of chronic myeloid leukemia. MAPK signaling pathway, extracellular matrix (ECM–receptor interaction was found tightly associated with EBFs determined risk in GC patients. Hou et al. [56] found that the EBF1 gene suppresses the activation of MAPK pathway, and promotes the cell proliferation and migration of bone marrow CD34^+^ cells, but inhibits the cell apoptosis. Interestingly, Kong et al. [57] also demonstrated that the p38 MAPK pathway could activate MEF2C to drive B-cell differentiation, MEF2C is a co-regulator of EBF1 to impact B cell-specific transcription.

## Conclusion

We evaluated the single and integrative prognostic value of EBFs in GC patients, the increased EBFs determined risk score linked with the poor prognosis and shorter OS. The mechanism of EBFs impacting GC tumorigenesis might realize through MAPK and extracellular matrix pathways. Our new findings provide a novel insight into the prediction of prognosis and clinical treatment of GC patients.

## Supplementary Material

Supplementary Table S1

## Data Availability

The data used to support the findings of the present study are included within the article. The gene expression data can be accessed on TCGA and GEO.

## Abbreviations

DEG: different expression gene
EBF: early B-cell factor
ECM: extracellular matrix
GC: gastric cancer
GEO: Gene Expression Omnibus
KEGG: Kyoto Encyclopedia of Genes and Genomes
OS: overall survival
PD-1: programmed cell death-1
PD-L1: programmed death ligand-1
PD-L2: programmed death ligand-2
ssGSEA: single-sample gene set enrichment analysis
STAD: stomach adenocarcinoma
TCGA: The Cancer Genome Atlas
