# Supplementary material for: Early B-cell factors involve in the tumorigenesis and predict the overall survival of gastric cancer
Source: Biosci Rep. 2021 Jun 28;41(7):BSR20210055. doi: 10.1042/BSR20210055 (PMC8239495; doi:10.1042/BSR20210055)
Supplement: Supplementary Table S1 [file BSR-2021-0055_supp.pdf]

Table S1. The correlations between 28 immunotypes and four EBFs.

cell type	EBF1		EBF2		EBF3		EBF4	
	correlation	p value	correlation	p value	correlation	p value	correlation	p value
Activated B cell	0.456	< 0.001	0.127	0.01	0.361	< 0.001	0.172	< 0.001
Activated CD4 T cell	< 0.001	0.17	< 0.001	0.003	< 0.001	0.015	< 0.001	< 0.001
Activated CD8 T cell	0.161	0.001	< 0.001	0.942	0.107	0.03	< 0.001	0.007
Activated dendritic cell	0.15	0.002	0.034	0.493	0.142	0.004	< 0.001	0.001
CD56bright natural killer cell	< 0.001	0.007	< 0.001	< 0.001	< 0.001	0.094	< 0.001	0.46
CD56dim natural killer cell	< 0.001	0.536	< 0.001	0.144	< 0.001	0.447	0.045	0.361
Central memory CD4 T cell	0.546	< 0.001	0.341	< 0.001	0.482	< 0.001	0.263	< 0.001
Central memory CD8 T cell	0.292	< 0.001	0.279	< 0.001	0.319	< 0.001	< 0.001	0.772
Effector memeory CD4 T cell	0.581	< 0.001	0.475	< 0.001	0.519	< 0.001	0.149	0.002
Effector memeory CD8 T cell	0.399	< 0.001	0.234	< 0.001	0.369	< 0.001	0.087	0.078
Eosinophil	0.47	< 0.001	0.226	< 0.001	0.363	< 0.001	0.105	0.032
Gamma delta T cell	0.174	< 0.001	0.13	0.008	0.168	< 0.001	< 0.001	0.033
Immature B cell	0.464	< 0.001	0.205	< 0.001	0.383	< 0.001	0.106	0.031
Immature dendritic cell	0.328	< 0.001	0.27	< 0.001	0.353	< 0.001	0.172	< 0.001
Macrophage	0.483	< 0.001	0.348	< 0.001	0.451	< 0.001	0.211	< 0.001
Mast cell	0.598	< 0.001	0.439	< 0.001	0.546	< 0.001	0.257	< 0.001
MDSC	0.385	< 0.001	0.254	< 0.001	0.356	< 0.001	0.072	0.145
Memory B cell	0.089	0.069	0.088	0.075	0.077	0.12	< 0.001	0.004
Monocyte	0.172	< 0.001	0.005	0.921	0.152	0.002	0.108	0.027
Natural killer cell	0.592	< 0.001	0.513	< 0.001	0.56	< 0.001	0.247	< 0.001
Natural killer T cell	0.488	< 0.001	0.411	< 0.001	0.466	< 0.001	0.201	< 0.001
Neutrophil	< 0.001	0.349	< 0.001	0.242	< 0.001	0.313	< 0.001	0.007
Plasmacytoid dendritic cell	0.719	< 0.001	0.543	< 0.001	0.661	< 0.001	0.424	< 0.001
Regulatory T cell	0.421	< 0.001	0.343	< 0.001	0.409	< 0.001	0.098	0.047
T follicular helper cell	0.517	< 0.001	0.4	< 0.001	0.509	< 0.001	0.219	< 0.001
Type 1 T helper cell	0.487	< 0.001	0.285	< 0.001	0.433	< 0.001	0.133	0.007
Type 17 T helper cell	< 0.001	< 0.001	< 0.001	< 0.001	< 0.001	< 0.001	< 0.001	< 0.001
Type 2 T helper cell	0.358	< 0.001	0.309	< 0.001	0.342	< 0.001	0.095	0.055
